# The Relationships of Experiencing Workplace Bullying with Mental Health, Affective Commitment, and Job Satisfaction: Application of the Job Demands Control Model

**DOI:** 10.3390/ijerph17062151

**Published:** 2020-03-24

**Authors:** Nicole M. Steele, Bryan Rodgers, Gerard J. Fogarty

**Affiliations:** 1 School of Demography, Australian National University, Canberra, ACT 0200, Australia; bryan.rodgers@anu.edu.au; 2 Division of Research and Innovation, University of Southern Queensland, Toowoomba, QLD 4350, Australia; gerry.fogarty@usq.edu.au

**Keywords:** bullying, mobbing, psychological distress, commitment, satisfaction, military

## Abstract

There have been very few theoretical models published to understand the relationship between workplace bullying and different outcome variables. Applying the Job Demands Control (JDC) model, this study analyzed workplace bullying alongside ‘traditional’ job stressors of role overload and low job control to determine the relative associations of each with mental health and wellbeing. These relative associations have not been well documented. Data were obtained from an organizational climate questionnaire administered to 21 Australian Defence Force units (*n* = 3193). Results indicated that the correlations between bullying and psychological distress (*r* = 0.39), job satisfaction (*r* = −0.28), and affective commitment (*r* = −0.22) were all significant and for some outcomes greater than those involving the traditional job stressors. Furthermore, for each of these three outcomes, bullying contributed incremental variance after controlling for other job demands. These results support earlier claims that workplace bullying requires the same attention given to traditional work stressors. The JDC model provides a strong theoretical base to investigate workplace bullying. Testing against other stressors allows for consideration of the broader context of workplace bullying when managing the workforce.

## 1. Introduction

Bullying is arguably one of the most pervasive stressors to be experienced at work [1,2,3]. Definitions of workplace bullying have varied depending on the research perspective or professional interest [4,5]. At its most basic level, workplace bullying is when unreasonable negative acts towards a person or group of people are both persistent and repeated, creating a risk to the health and safety of individuals [6]. 

A meta-analysis of studies conducted across almost 25 years reported that workplace bullying had positive associations with anxiety (*r* = 0.27), depression (*r* = 0.34), and post-traumatic stress symptoms (*r* = 0.37), and negative associations with job satisfaction (*r* = −0.22) and organizational commitment (*r* = −0.19) [7]. Longitudinal studies have also found bullying to be significantly associated with depression, poor general health, and sleep disturbance up to four years after the initial exposure to bullying [8]. Exposure to workplace bullying has been shown to increase the risk of sickness absence (odds ratio 1.58, 95% CI 1.39–1.79) [9] and suicidal ideation (odds ratio 2.05, 95% CI 1.08–3.89) [10].

Even with conservative prevalence rates, at least one in ten employees within an organization experiences workplace bullying at any given point in time [11]. Workplace bullying often has a long duration. Typically, bullying occurs over one to two years [12], with some studies reporting durations of four years or more [8,12]. Due to its prevalence, duration, and widespread impact, organizations that have not addressed workplace bullying stand to lose billions of dollars through consequences such as lost productivity, absenteeism, and turnover [13,14].

A meta-analysis of research into the consequences of workplace bullying indicated that there have been very few theoretical models published to understand the relationship between workplace bullying and different outcome variables [7]. Similarly, after reviewing the literature published on workplace bullying from 1990 to 2016, Rai and Agarwal [15] concluded that the theoretical development of workplace bullying ‘has been…weak’ (p. 33).

As a severe source of workplace stress, we proposed to underpin our hypotheses and investigate relationships of workplace bullying using the well-validated Job Demands Control (JDC) model of occupational stress [16,17]. A common feature of occupational stress theories is that experiencing workplace stressors generates negative physical, psychological, or behavioural changes in the individual [18], although the strength of the relationships between these workplace stressors and mental health and wellbeing varies [19,20]. In a review of over 30 studies exploring the strain hypothesis within the JDC model, almost two thirds demonstrated support for the negative relationship between job demands and mental health (anxiety, depression, and psychological distress), and just over one half of the studies found partial or full support for the negative associations between job demands and job satisfaction [21]. Studies applying the JDC model have demonstrated that high job demands and low job control result in low affective commitment to the organization, either directly [22] or indirectly through exhaustion and cynicism [23]. 

Many applications of occupational stressor-strain models have treated workplace bullying as an outcome variable [24,25,26,27]. By applying the strain hypothesis within the JDC model, we first aimed to treat workplace bullying as a job demand and to analyze this stressor alongside ‘traditional’ job stressors of role overload and low job control. This application allows for an assessment of the relative associations of each potential stressor with the mental health and wellbeing of employees. Through this aim we assessed if exposure to workplace bullying explained variance in psychological distress, affective commitment, and job satisfaction after adjusting for the influence of role overload and low job control. Testing against other job stressors allows for consideration of the broader context of workplace bullying when developing workforce plans, strategies, and interventions. 

The relative effect of workplace bullying compared to other frequently encountered job stressors has not been well documented [1]. In a meta-analysis, Bowling and Beehr [28] reported that workplace harassment was negatively associated with job satisfaction, organizational commitment, and wellbeing after accounting for role ambiguity and role conflict. Given the broad definition of workplace harassment—inclusive of abusive supervision, petty tyranny, interpersonal conflict, workplace bullying, and harassment—the contribution of workplace bullying to these findings was unclear. Using a narrower definition, Hauge et al. [1] found that workplace bullying accounted for variation in anxiety, depression, job satisfaction, turnover intention, and absenteeism beyond what was accounted for by job demands, decision authority, role ambiguity and role conflict. We build upon the work of Hauge et al. [1] by adding an affective commitment scale to their measures to test the relative association of workplace bullying compared to role overload and low job control. While often thought to be inherent to the demands of the job, we proposed that role overload and job control can be manipulated by employees at different levels in an organization—subordinate, superior or coworker. Employees at these levels can also exhibit behaviours associated with workplace bullying to create stress in others. As such, we predicted that workplace bullying would operate similarly to demands traditionally examined under the JDC model, and made the following hypotheses:

**Hypothesis** **1.** *Experiencing workplace bullying would be negatively associated with affective commitment and job satisfaction, and positively associated with psychological distress*.

**Hypothesis** **2.** *Experiencing workplace bullying would explain variance in psychological distress after adjusting for the influence of role overload and job control*.

**Hypothesis** **3.** *Experiencing workplace bullying would explain variance in affective commitment after adjusting for the influence of role overload and job control*.

**Hypothesis** **4.** *Experiencing workplace bullying would explain variance in job satisfaction after adjusting for the influence of role overload and job control*.

The second aim of this study was to determine the strength of the relationships of workplace bullying with psychological distress, affective commitment, and job satisfaction, after accounting for sex, age, role overload, and job control. The following hypothesis was made:

**Hypothesis** **5.** *After accounting for sex, age, role overload, and job control, there would be a dose effect between frequency of experiencing workplace bullying and low affective commitment, low job satisfaction, and psychological distress*.

## 2. Materials and Methods 

### 2.1. Procedure and Participants

This study was based on data collected through an organizational climate questionnaire, administered to units upon request by the Commanding Officer [29]. The scales in the questionnaire measured the main components of the JDC model as well as bullying behaviour. The anonymous nature of the questionnaire, coupled with administration by an organization separate from the chain of command, provided assurance to respondents that results would be confidential. The aim of this method was to encourage more accurate reflections on a sensitive topic such as workplace bullying than otherwise would have been provided should respondents be identified. In addition, there a risk that due to stigma, misinterpretation, or tolerance and acceptance, employees may experience extreme negative behaviours but not report these experiences as being ‘bullied’. The method of presenting a list of specific negative behaviours for respondents to report against reduces this risk. 

All 21 Australian Defence Force (ADF) units who requested a climate questionnaire during 2011 and 2012 were included in the final sample. These units were dispersed across Australia. The questionnaire was administered to 17 units via face-to-face group administration, and 4 units via mail administration. 

Response rates across the 21 units ranged from 31% to 79%, with an average response rate of 55%. This response rate was deemed acceptable given the operational tempo of the units surveyed, with personnel absent from the workplace due to deployment-related leave, training exercises, courses, and personal leave. There was minimal difference in response rates between the face-to-face administration and the mail-out administrations.

Participants included 3193 military and civilian personnel. Most personnel had served with their current unit for over 17 months and were within their first 9 years of service (56.5%). Members of the Permanent Force (80.3%) and Non-Commissioned Officers (76.1%) comprised the majority of the sample. The breakdown of gender, rank, and work status aligned with rates in the broader ADF at the time of the study [30]. Generalization of prevalence findings to the broader ADF is not possible due to the small numbers of units analyzed, and relatively low number of respondents, especially from the Navy and Air Force (6% and 20% of the sample, respectively). 

Ethical approval of the study was obtained from the Australian Defence Human Research Ethics Committee (Protocol 618_11) and the Australian National University Human Research Ethics Committee (Protocol 350_11). 

### 2.2. Scales and Measures

#### 2.2.1. Role Overload 

The 3 items within the Role Overload scale were derived from the Occupational Roles Questionnaire (ORQ) within the Occupational Stress Inventory-Revised [31]. The ORQ measures the presence of stressors related to the nature of the individual’s role within an organization. The original Role Overload subscale had ten items. The three items of the Role Overload scale included in this study were: “At work I am expected to do too many different tasks in too little time”, “I am expected to do more work than is reasonable”, and “I work under tight deadlines”. The Cronbach’s alpha coefficient for the scale was 0.81. 

Participants were asked to rate the frequency with which they experienced each role overload item on a five-point Likert scale (1 = never, 2 = rarely, 3 = sometimes, 4 = frequently, 5 = always). Higher mean scores indicated that participants often experienced role overload in the workplace.

#### 2.2.2. Job Control

Two of the items assessing job control were originally part of a broader six-dimension Organizational Climate Questionnaire [32] used within Canada’s Department of National Defence to examine Officer Cadets’ perceptions of the organizational climate at the Royal Military College of Canada [32]. A further three items assessing job control were developed by psychologists within the Australian and Canadian Defence Forces (A. Twomey, personal communication, June 20, 2011). The five items were:

1. I can make important decisions without having to ask permission from my immediate supervisor.
2. The unit lets me do work according to my own judgment.
3. The unit allows me to show initiative.
4. The unit treats me as a responsible person.
5. The unit trusts me to do my work.

The Cronbach’s alpha coefficient for the five job control items was 0.86. Personnel were asked to respond to the five job control items by indicating agreement on a five-point Likert scale (1 = strongly disagree to 5 = strongly agree). High mean scores indicated high perceived job control. 

#### 2.2.3. Psychological Distress

Psychological distress was measured by the Kessler 10 [33]. The K10 is a 10-item self-report measure of non-specific psychological distress in the areas of depression and anxiety [33,34]. The Cronbach’s alpha coefficient for the K10 was 0.91. Personnel respond to a series of ten questions by indicating on a five-point Likert scale (1 = none of the time, 2 = a little of the time, 3 = some of the time, 4 = most of the time, 5 = all of the time) how they have been feeling over the past four weeks. Scores on each item are added to yield a K10 total score, with a high score indicating high levels of psychological distress.

#### 2.2.4. Affective Commitment

Affective commitment was measured using an abbreviated version of the 8-item Allen and Meyer’s [35] Affective Commitment Scale. Whilst each item referred to commitment to the ADF/Defence, respondents were primed by the statements: ‘If you are in the ADF, consider how you feel about your specific Service (Navy, Army, or Air Force). If you are civilian, consider how you feel about the Department of Defence.’ The items included in this scale were “The ADF has a great deal of personal meaning for me”, “I enjoy discussing ADF/Defence with people outside it”, and “I feel a sense of belonging to the ADF”. The Cronbach’s alpha coefficient for this scale was 0.80. Respondents were asked to rate their level of agreement with each statement on a five-point Likert scale (1 = strongly disagree to 5 = strongly agree). Higher mean scores indicated higher affective commitment.

#### 2.2.5. Job Satisfaction

The 28-item Job Satisfaction scale within the organizational climate questionnaire has the capacity to assess both overall job satisfaction (3 items) and satisfaction with particular facets of military service (25 items). The scale was based on Spector’s [36] Job Satisfaction Survey (JSS) and Hackman and Oldham’s [37] Job Diagnostic Survey (JDS). The 3 items measuring overall job satisfaction were dispersed throughout the 28-item scale. The items were “I am satisfied with my current job”, “I like doing the things I do at work”, and “I am satisfied with the kind of work I do in my current job”. The Cronbach’s alpha coefficient for these three items was 0.90. Respondents were asked to rate their level of agreement with each statement on a five-point Likert scale (1 = strongly disagree to 5 = strongly agree). Higher mean scores indicated higher job satisfaction.

#### 2.2.6. Workplace Bullying

The six workplace bullying items assessed:

1. Physical violence or threats of physical violence.
2. Excessive criticism.
3. Deliberate exclusion from social gatherings.
4. Humiliating comments.
5. Damaging rumours/gossip.
6. Deliberate withholding of equipment, resources, or information.

The items selected for inclusion by the first author had been assessed in a climate questionnaire within a similar large government organization in Australia [38]. The Cronbach’s alpha coefficient for the workforce bullying scale was 0.85. 

Respondents were asked to rate the frequency of experiencing each behaviour over the past 6 months on a five-point Likert scale (1 = Never; 2 = Rarely (e.g., once or twice); 3 = Sometimes (e.g., once a month); 4 = Often (e.g., 4–5 times a month); 5 = Very Often (e.g., 2–3 times a week)). Depending upon the analysis, the measure of workplace bullying in this study is treated in two ways:

A continuous measure of exposure to workplace bullying: the score of the six workplace bullying items (hereafter Bullying score) is summed. The Bullying score ranges from 6 to 30, with higher scores indicating higher frequency of experiencing workplace bullying.

An ordinal measure with four categories formed from the Bullying score: ‘Never’ (Bullying score of 6, 59.4% of sample); ‘Rarely’ (Bullying score of 7–8, 17.7% of sample); ‘Sometimes’ (Bullying score of 9–12, 14.0% of sample); and ‘Often’ (Bullying score of 13–30, 8.9% of sample).

### 2.3. Statistical Analyses

All statistical analyses were conducted using IBM SPSS version 20 ((SPSS Inc., Chicago, IL, USA) [39]. Correlational analyses tested the associations of experiencing workplace bullying with psychological distress, affective commitment, and job satisfaction (H1). Due to the significant skewness of two variables, Workplace Bullying and Psychological Distress, both Pearson’s product moment correlation coefficients and Spearman’s rho correlations were estimated.

To test H2 to H4, three hierarchical multiple linear regressions were conducted, with psychological distress, affective commitment, or job satisfaction as the dependent variable (DV). In Step 1, age and gender were entered. In Step 2, role overload and job control were added, and finally in Step 3 workplace bullying was entered. Step 3 determined the variance accounted for by workplace bullying, after adjustment for role overload and job control. The three two-way interactions between role overload, job control, and workplace bullying were also tested for each DV (i.e., psychological distress, affective commitment, and job satisfaction). No interaction was significant. 

To estimate the strength of associations between experiencing workplace bullying and each of the DVs (H5), standardized residuals were calculated from linear regression models, taking account of four covariates (age, gender, role overload, and job control). 

As well as their use as continuous measures, the standardized residuals were dummy coded. For job satisfaction and affective commitment, the 1/8th largest negative residuals were coded ‘1’ (i.e., low satisfaction and commitment after adjustment for the covariates) and the remaining residuals were coded ‘0’. For psychological distress, the 1/8th largest positive residuals were coded ‘1’ (i.e., high distress after adjustment for covariates) and the remaining residuals were coded ‘0’. The percentage of large residuals was tabulated against the four frequency groups of bullying (never, rarely, sometimes, often) to show the relationships between frequency of bullying and the adjusted outcomes.

Curve fitting further determined the nature of these associations (i.e., linear or quadratic) using the continuous measures of the residuals. These analyses were conducted using the SPSS curve estimation module.

## 3. Results

### 3.1. Aim 1. H1. Experiencing Workplace Bullying Would be Negatively Associated with Affective Commitment and Job Satisfaction, and Positively Associated with Psychological Distress

In Table 1, Pearson’s product moment correlation coefficients are shown below the diagonal, with Spearman’s rho correlations reported above the diagonal. Cronbach’s alpha coefficients are on the diagonal. All study variables showed good to excellent Cronbach’s alpha coefficients [40].

Both parametric and non-parametric correlations showed predominantly moderate correlations [41] for workplace bullying with all outcome measures. The association between workplace bullying and psychological distress was the strongest (*r* = 0.39), followed by job satisfaction (*r* = −0.28) and affective commitment (*r* = −0.22). These relationships were in the hypothesized direction: as the experience of workplace bullying occurred more frequently, psychological distress increased, and affective commitment and job satisfaction decreased (providing support for Hypothesis 1). With the exception of a stronger association between workplace bullying and psychological distress, job control showed greater associations with each outcome, followed by workplace bullying, and then role overload. 

### 3.2. Aim 1. H2. Experiencing Workplace Bullying Would Explain Variance in Psychological Distress after Adjusting for the influence of Role Overload and Job Control

The second hypothesis was tested through hierarchical regression. Table 2 details the results of this analysis. Due to the possible differences across gender and age for reported psychological distress [42,43] these variables were entered first. The results of Step 1 indicated that the variance accounted for (*R*^2^) by gender and age was small (0.01). In Step 2, role overload and job control were entered into the regression equation. The change in variance (Δ*R*^2^ = 0.18) was statistically significant (*p* < 0.01). In Step 3, bullying was entered and the change in variance (Δ*R*^2^ = 0.05) was again significant (*p* < 0.01). In the final model, all variables contributed significantly to predicting psychological distress. Workplace bullying (β = 0.26) showed the strongest association with psychological distress. The final model explained 24% of the variance (*F*(5, 3014) = 191.26, *p* < 0.01). Taken together these findings support Hypothesis 2.

### 3.3. Aim 1. H3. Experiencing Workplace Bullying Would Explain Variance in Affective Commitment after Adjusting for the Influence of Role Overload and Job Control

Table 3 details the results of the hierarchical regression with affective commitment as the DV. Again, due to possible age and gender differences on reported affective commitment [44,45] these variables were entered first. The results of this first step indicated gender and age accounted for 0.04 of the variance (*p* < 0.01). In Step 2, role overload and job control were entered into the regression equation. The change in variance (Δ*R*^2^ = 0.09) was significant (*p* < 0.01). In Step 3, workplace bullying was entered and explained very small, yet significant, additional variance (Δ*R*^2^ = 0.003). In the final model, age, job control, and workplace bullying contributed significantly to the prediction of affective commitment (*F*(5, 3011) = 88.43, *p* < 0.01). The final model explained 13% of the variance in affective commitment. Regression results showed that workplace bullying had a stronger association with affective commitment (β = −0.07) but not with role overload (β = −0.02). Job control had the largest beta value (β = 0.28), indicating that a difference in job control would be associated with the largest difference in affective commitment. While the findings support Hypothesis 3, it is noted that experiencing workplace bullying did not contribute much to the variance in affective commitment after adjustment for role overload and job control.

### 3.4. Aim 1. H4. Experiencing Workplace Bullying Would Explain Variance in Job Satisfaction after Adjusting for the Influence of Role Overload and Job Control

Table 4 details the results of the hierarchical regression with job satisfaction as the dependent variable. Due to possible age and gender differences in reported job satisfaction [46,47] these variables were entered first. The results of this first step indicated that the variance accounted for (R2) was 0.02 (*p* < 0.01). In Step 2, role overload and job control were entered into the regression equation. The change in variance (Δ*R*^2^ = 0.24) was statistically significant (*p* < 0.01*).* In Step 3, workplace bullying was entered and change in variance was small (Δ*R*^2^ = 0.01) but significant (*p* < 0.01). In the final model all variables, except gender, contributed significantly to the explanation of job satisfaction, with the final model explaining 27% of the variance (*F*(5, 3028) = 219.81, *p* < 0.01).

Of all the predictors, job control had the strongest association with job satisfaction (β = 0.45). The associations of workplace bullying (β = −0.06) and role overload (β = −0.12) with job satisfaction were significant, though not as strong as that shown for job control. Although Hypothesis 4 was supported, the experience of being bullied at work did not explain much variance in job satisfaction after adjusting for role overload and job control.

### 3.5. Aim 2. H5. After Accounting for Sex, Age, Role Overload, and Job Control, There Would Be a Dose Effect between Frequency of Experiencing Workplace Bullying and Low Affective Commitment, Low Job Satisfaction, and Psychological Distress

**Figure 1:**
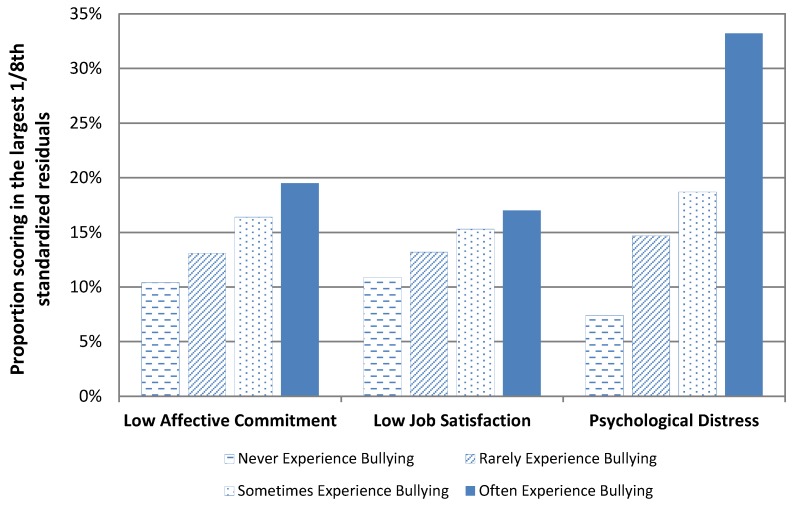
Relationships between frequency of experiencing workplace bullying and standardized residuals of (low) affective commitment, (low) job satisfaction, and (high) psychological distress.

Figure 1 shows the strong positive relationships between the frequency of experiencing workplace bullying and the adjusted measure of each outcome. These relationships demonstrate a clear dose effect and support for Hypothesis 5: as the frequency of experiencing bullying increases so too do the reported negative effects. This association is particularly strong between workplace bullying and psychological distress. Respondents who often experienced workplace bullying were four times more likely to have large psychological distress residuals compared to those never experiencing bullying. 

Curve fitting determined whether linear or quadratic functions better accounted for the relationships between workplace bullying and adjusted measures of psychological distress, affective commitment, and job satisfaction. Tests showed that linear equations adequately fitted the relationships between each outcome measure and workplace bullying, and that quadratic functions did not improve the fit significantly (psychological distress (*F*(1, 3018) = 162.84, *p* < 0.01; *R*^2^ = 0.05), affective commitment (*F*(1, 3015) = 9.16, *p* < 0.01; *R*^2^ = 0.003), job satisfaction (*F*(1, 3027) = 10.84, *p* < 0.01; *R*^2^ = 0.004)).

## 4. Discussion

Previous applications of the JDC model have shown that traditional job stressors of role overload and low job control are directly associated, to varying degrees, with mental health, job satisfaction, and commitment. The JDC model provided a strong theoretical base to investigate social stressors such as workplace bullying. 

To address the study’s first aim, the strain hypothesis of the JDC model was applied to workplace bullying and we found that when compared alongside two other job stressors, the associations between experiencing workplace bullying and outcomes were similar in magnitude, and for some consequences even stronger, than the reported associations of experiencing role overload and low job control. No significant interactions between workplace bullying and the other two job stressors were found across all outcomes. 

Exploring multiple workplace stressors allowed for a relative comparison of each association with commitment, job satisfaction and psychological distress. Overall, job control had greater associations with these outcomes, followed by workplace bullying, and then role overload. The exception was the stronger association between workplace bullying and psychological distress. When compared to other stressors, a change in workplace bullying would see the largest change in mental health. Workplace bullying also explained significant variance in each outcome after adjusting for other job demands, demonstrating support for Hypotheses 1–4. 

Findings from this study’s second aim showed that employees who often experienced workplace bullying were four times more likely to report high psychological distress compared to those never experiencing bullying. Similar patterns, though not as strong, were shown for low commitment and job satisfaction. There was support for Hypothesis 5. Hauge et al. [1] also reported that workplace bullying had stronger associations with mental health than with other outcomes such as turnover intentions and job satisfaction. Similarly, in Hauge et al.’s [1] study, workplace bullying showed a substantial relative contribution in relation to mental health, with smaller contributions identified for job satisfaction, turnover intention and absenteeism. The relationship between experiencing workplace bullying and each outcome was demonstrated after accounting for other, potentially confounding, variables. It was shown that even when bullying was experienced rarely (e.g., once or twice), there was a strong association with poor commitment, job satisfaction, and mental health.

In comparison to research on traditional role stressors, research into workplace bullying is a relatively new field, dating back to the early 1990s [48,49]. This study demonstrated the importance of now including workplace bullying into the taxonomy of workplace stressors when conducting research or addressing workplace climate. The JDC model, traditionally applied to workplace stressors such as role overload and job control, should also be applied to social stressors such as workplace bullying. The associations between experiencing workplace bullying and three key outcomes of the JDC model were all significant and showed the same direction of associations with mental health and wellbeing as traditional workplace stressors. After adjusting for other variables, workplace bullying had significantly strong associations with all outcomes and showed a clear dose effect between the frequency of workplace bullying and psychological distress, affective commitment, and job satisfaction. Even a rare experience of being bullied was associated with poorer outcomes. Given the high prevalence of bullying in the workplace and its duration, our findings on its associations with key outcomes indicate the importance of its contribution to the JDC model.

Current findings from the application of the JDC model provide practical implications for organizations. Even if autonomy and role overload are addressed, employee engagement and satisfaction will be poorer for those employees experiencing workplace bullying. As this study showed, even at low frequency, bullying was associated with poorer mental health and wellbeing. Similarly, even if employees are provided with autonomy in decision making and are able to moderate the flow of work, they may experience psychological distress if they are targets of workplace bullying.

Including workplace bullying alongside other workplace stressors allows for consideration of a wider range of job demands that an employee may experience at work. A recent example of this in practice is the Royal Australian Air Force’s climate survey, the Snapshot. In this climate tool, workplace bullying is assessed alongside other work-related stressors such as role overload, to provide a more comprehensive assessment of job demands [50].

Workplace design, planning, and strategies have tended to focus on traditional job stressors, but their effectiveness would be enhanced if consideration is also given to the social stressors that arise from workplace interactions. Recruiting the right employees to meet the strategic goals of an organization is important, but so too is ensuring that these employees remain engaged, productive, and satisfied. As shown in this study, workplace bullying has a significant association with these outcomes and requires due consideration in any strategic planning activity. 

### 4.1. Limitations

There are several limitations to this study. This study was cross-sectional and did not use an experimental design, and as a result no cause-and-effect findings can be made. Only targets’ perception of the occurrence of workplace bullying were captured. Poor record keeping practices of formal complaints of workplace bullying in the ADF [51] at the time this study commenced made it difficult to obtain other sources of information on workplace bullying. However, due to stigma and other barriers, targets may not come forward to formalize complaints of workplace bullying. Therefore, data on formal investigations of workplace bullying may underrepresent the occurrence of bullying in the workplace. While a substantial proportion of those experiencing unacceptable behaviours may be missed if only formal complaint data are analyzed, complementing this data with other sources of information, such as through surveys or interviews with targets, assists in building the picture of workplace bullying in an organization. This study did not seek to interview or collect information from perpetrators. Due to the sensitive nature of the research, and social desirability, there is often reluctance on the part of perpetrators to identify themselves as such in this type of research [52,53].

The selection of units for inclusion in this study was limited to those units whose commanders requested an assessment of organizational climate. Opting in to the process may have restricted the range of results for some variables, although findings indicated that there was wide variability in workplace climates, especially in perceptions of workplace bullying, and health and wellbeing outcomes. Units with potential issues and units with better workplace climates participated.

This research was conducted with a military sample, potentially limiting the generalization of results to that of other male-dominated hierarchical organizations such as the religious institutions, medical, police and emergency services. There is limited published research on workplace bullying in the military—to date, there has been no empirical research published in the ADF on the impact of workplace bullying. Yet, there is widespread research that has been published in civilian organizations, companies that are neither male-dominated nor hierarchical, that align with the findings in this study. Similarly, Campbell and Nobel [54] note that occupational stressors experienced by military personnel overlap substantially with those found in civilian workplaces. This is particularly so in a garrison (non-operational) environment - the setting of this study. It is therefore plausible that the relationships between workplace bullying and the mental health and wellbeing of ADF personnel would generalize to civilian workplaces. 

### 4.2. Future Research 

Future studies would be strengthened if the subjective data collected through questionnaires were coupled with (1) interview data from targets and if possible from perpetrators, and (2) with formal complaint data gained through records of formal investigations into workplace bullying. This approach needs to be cautioned with the knowledge that targets of bullying do not always report the occurrence. 

This study applied the JDC model to analyze the consequences of workplace bullying, adding to research that has explored the antecedents to negative workplace behaviours [55,56,57]. Future research would benefit from further applications of occupational stressor-strain models to analyze both antecedents and consequences of workplace bullying. 

There has been a paucity of studies exploring workplace bullying at the individual and group/organization/society level [58]. Findings from this study suggest that various occupational stressors occur simultaneously at work. Organizational climates high in bullying have been shown to have poor leadership, lack of role clarity, high role ambiguity, and poor working conditions [57,59,60,61]. Exploring workplace bullying at the organizational level would contribute substantially to what is already known at the individual level. Further research at different levels would enable organizations to develop strategies to address workplace climates that enable or perpetuate bullying [62].

## 5. Conclusions

The JDC model provides a strong theoretical base to investigate workplace bullying. Findings indicated that the correlations between bullying and psychological distress, job satisfaction, and affective commitment were all significant and for some outcomes greater than those involving the traditional job stressors. Furthermore, for each of these three outcomes, bullying contributed incremental variance after controlling for other job demands. Testing against other job stressors allows for consideration of the broader context of workplace bullying and the extent to which it occurs with other job stressors/demands. Understanding the associations of experiencing these job stressors with mental health and wellbeing helps inform workplace design strategies and addresses areas of organizational climate to ensure employees remain engaged, motivated, and satisfied while working in mentally-healthy workplaces.

## Figures and Tables

**Table 1:** Descriptives, reliabilities and correlations among study variables.

	Correlations							
Variable	Mean	SD	1	2	3	4	5	6
1. Workplace Bullying	7.81	3.47	0.85	0.20 **	−0.36 **	0.36 **	−0.18 **	−0.27 **
2. Role Overload	3.05	0.79	0.19 **	0.81	−0.04 *	0.27 **	−0.01	−0.15 **
3. Job Control	3.71	0.75	−0.40 **	−0.08 **	0.86	−0.32 **	0.33 **	0.48 **
4. Psychological Distress	15.22	6.20	0.39 **	0.26 **	−0.35 **	0.91	−0.21 **	−0.36 **
5. Affective Commitment	3.72	0.85	−0.22 **	−0.04 *	0.34 **	−0.25 **	0.80	0.41 **
6. Job Satisfaction	3.52	0.95	−0.28 **	−0.15 **	0.50 **	−0.40 **	0.45 **	0.90

Note. Spearman’s rho correlations are above the diagonal, Pearson’s correlations are below the diagonal. Cronbach’s alpha coefficients are on the diagonal. *n* range = 3163–3193. * *p* < 0.05. ** *p* < 0.01.

**Table 2:** Summary of hierarchical regression analysis for variables predicting psychological distress (*n* = 3020).

	Model 1				Model 2				Model 3			
Variable	*B*	*SE B*	β	*sr* ^2^	*B*	*SE B*	β	*sr* ^2^	*B*	*SE B*	β	*sr* ^2^
Gender	0.28	0.30	0.02	0.00	0.64	0.27	0.04 **	0.00	0.73	0.26	0.05 **	0.00
Age	−0.51	0.08	−0.12 **	0.01	−0.39	0.08	−0.09 **	0.01	−0.27	0.07	−0.06 **	0.00
Role Overload					2.01	0.13	0.26 **	0.06	1.65	0.13	0.21 **	0.04
Job Control					−2.56	0.14	−0.31 **	0.09	−1.80	0.15	−0.22 **	0.04
Bullying									0.46	0.03	0.26 **	0.05
*R* ^2^	0.01				0.19				0.24			
∆*R*^2^					0.18				0.05			
*F* for change in *R*^2^	20.77 **				325.83 **				206.34 **			

* *p* < 0.05. ** *p* < 0.01.

**Table 3:** Summary of hierarchical regression analysis for variables predicting affective commitment (*n* = 3016).

	Model 1				Model 2				Model 3			
Variable	*B*	*SE B*	β	*sr* ^2^	*B*	*SE B*	β	*sr* ^2^	*B*	*SE B*	β	*sr* ^2^
Gender	−0.04	0.04	−0.02	0.00	−0.06	0.04	−0.03	0.00	−0.06	0.04	−0.03	0.00
Age	0.11	0.01	0.19 **	0.04	0.07	0.01	0.13 **	0.01	0.07	0.01	0.12 **	0.01
Role Overload					−0.04	0.02	−0.04 *	0.00	−0.03	0.02	−0.02	0.00
Job Control					0.33	0.02	0.30 **	0.08	0.31	0.02	0.28 **	0.06
Bullying									−0.02	0.01	−0.07 **	0.00
*R* ^2^	0.037				0.125				0.128			
∆*R*^2^					0.09				0.00			
*F* for change in *R*^2^	58.50 **				150.29 **				11.49 **			

* *p* < 0.05. ** *p* < 0.01.

**Table 4:** Summary of hierarchical regression analysis for variables predicting job satisfaction (*n* = 3028).

	Model 1				Model 2				Model 3			
Variable	*B*	*SE B*	β	*sr* ^2^	*B*	*SE B*	β	*sr* ^2^	*B*	*SE B*	β	*sr* ^2^
Gender	0.06	0.05	0.02	0.00	0.01	0.04	0.00	0.00	0.01	0.04	0.00	0.00
Age	0.10	0.01	0.15 **	0.02	0.04	0.01	0.06 **	0.00	0.03	0.01	0.05 **	0.00
Role Overload					−0.15	0.02	−0.13 **	0.01	−0.14	0.02	−0.12 **	0.01
Job Control					0.59	0.02	0.47 **	0.21	0.56	0.02	0.45 **	0.16
Bullying									−0.02	0.01	−0.06 **	0.00
*R* ^2^	0.02				0.26				0.27			
∆*R*^2^					0.24				0.01			
*F* for change in *R*^2^	36.93 **				491.58 **				13.58 **			

* *p* < 0.05. ** *p* < 0.01.
